# Review of the New Caledonian species of *Acritoptila* Wells, 1982 (Trichoptera, Insecta), with descriptions of 3 new species

**DOI:** 10.3897/zookeys.397.7059

**Published:** 2014-04-03

**Authors:** Alice Wells, Kjell Arne Johanson

**Affiliations:** 1 Australian Biological Resources Study, PO Box 787, Canberra, ACT 2601 Australia; 2 Department of Zoology, Swedish Museum of Natural History, Box 50007, SE-104 05 Stockholm, Sweden

**Keywords:** Spicipalpia, Hydroptilidae, New Caledonia, key

## Abstract

We review the New Caledonian representatives of the Australasian endemic hydroptiline genus *Acritoptila*, based on examination of a considerable collection of material in the Swedish Museum of Natural History and of types of previously established species. A key for identification of males is given and includes 3 species newly described here: *A. parallela* **sp. n.**, *A. forficata* **sp. n.** and *A. macrospina* **sp. n.** For all New Caledonian species, male genitalia are illustrated, and for 5 associated females, distinctive features are illustrated and described.

## Introduction

Among the microcaddisfly genera (Trichoptera: Hydroptilidae) found in the south-western Pacific region, several have restricted distributions whereas others are common also in the Oriental Region or are cosmopolitan. *Acritoptila* Wells, 1982 is one of those with a narrow distribution, first described for 3 Australian species (Wells 1982). Subsequently, another 2 Australian species were added by Wells (1990) and 7 species were described from New Caledonia by Kelley (1989) and Wells (1995). Two additional New Caledonian species were described by Oláh and Johanson (2010a), but below one of their names is considered a junior synonym, and 3 new species are described. Comparative notes and new records are given for previously established New Caledonian species. All specimens of *Acritoptila* have been collected at lights or in Malaise traps, none by sweep-netting.

The species in the genus *Acritoptila* were distinguished by Wells (1982) from those in the apparently related genera *Hellyethira* Neboiss, 1977, *Austratrichia* Wells, 1982 and *Mulgravia* Wells, 1982 on the basis of small but consistent differences in male inferior appendages. These include abdominal segment IX being sub-quadrate in ventral view, inferior appendages fused, and presence of a pair of spines, termed “parameres” by Wells (1982) when describing them in the south-western Australian species *Acritoptila globosa* Wells, 1982, but described by Kelley (1989) as spiny processes “projecting from the lateral margin of abdominal tergum X”. In Kelley’s illustrations of these “spiny processes” in lateral views of the male genitalia they arise from complex internal apodemes, from which appearance it is likely that they are moveable. But how closely associated they are with tergum X is open to conjecture. Recognition of homologies, and therefore assignment of terms to these and other processes in the often extraordinarily complex male genitalia of microcaddisflies is difficult. In the absence of evidence from developmental studies, putative homologies can be no more than hypotheses. It is difficult to decide, for example, how best to describe the suite of features seen in the male genitalia of *Acritoptila glossocercus* Kelley, 1989 (Figs 11, 12). The stout, dark, tapered setae apico-medially on the fused gonopods of this species may or may not be homologous with the rounded, knob-like setae seen in the Western Australian *Acritoptila globosa* and *Acritoptila margaretae* Wells, 1982 and in the New Caledonian *Acritoptila planichela* Kelley, 1989, *Acritoptila ouenghica* Wells, 1995, *Acritoptila macrospina* sp. n., and *Acritoptila parallela* sp. n. One set of species, with Australian and New Caledonian representatives, lacks the threadlike “parameres”, but has sclerotized processes laterally on tergite X. These are assumed homologues of the thread-like parameres.

In the context of congeners, meaningful description of structures in male genitalia of some of these species is difficult. Nevertheless, the New Caledonian species together with the 5 Australian species share the above small suite of genitalic features that suggest they form a monophyletic lineage. In contrast, distinctive female features are recognised for each of the few species that has been associated with males, such as the mesal elongate digitiform process on abdominal sternite VIII in *Acritoptila disjuncta* Kelley, 1989 (Fig. 24), pair of dark-tipped lobes on sternite VIII as in *Acritoptila crinita* Kelley, 1989 (Fig. 26), darkly pigmented mid ventral spine on abdominal sternite VIII in *Acritoptila chiasma* Kelley, 1989 (Fig. 27) and mid ventral glandular structure in *Acritoptila amphapsis* Kelley, 1989 (Fig. 28).

For most of the species described by Kelley, new illustrations are given here, drawn from fresh material and corroborated by examination of the holotypes. Final instar larvae have been associated for two Australian species (Wells 1990) and for two New Caledonian species (Figs 31–33) and have abdominal segments III to VIII swollen and segments I and II forming a narrow “waist”, superficially giving a appearance somewhat similar to the Hymenoptera petiole, a feature that distinguishes them from known larvae of *Hellyethira* (Wells 1985, 1997), which have the first 3 abdominal segments narrow. Several cases have been associated from pharate pupae and, similar to females, each is distinctive (Figs 30, 32, 34).

## Material and methods

The basis of this study is the collection of New Caledonia material made by K.A. Johanson (abbreviated throughout as KAJ) and associates from the Swedish Museum of Natural History, Stockholm, Sweden where most of the material is deposited; a small number of specimens, including several paratypes are deposited in the Australian National Insect Collection. All holotypes are lodged in the Muséum National d’Histoire Naturelle, Paris, France. Specimens were collected with light traps and Malaise traps. One of the authors (AW) examined holotypes of Kelley’s 6 species of *Acritoptila* deposited in the Bishop Museum in Honolulu, where they are stored as macerated specimens in glycerine in microvials.

Recently collected specimens were prepared for close study by maceration in KOH, then cleared in clove oil and mounted in Canada Balsam. Illustrations were prepared by methods described by Wells et al. (2013). A key is provided to adult males of New Caledonian species. Larvae and cases were associated from pharate adults.

Treatments of species are arranged in order such that those with most similar features are placed in close proximity. Terminology follows the recommendations of Oláh and Johanson (2010b), who argued for uniformity of terms across all Trichoptera taxa. Thus we have employed the terms “gonopods” and “subgenital processes” rather than “inferior appendages” and “subgenital plate”; these terms have been used in the two papers already published in this series of papers on New Caledonia Hydroptilidae (Wells and Johanson 2012; Wells et al. 2013).

### List of depository institutions with abbreviations used in the text

ANIC Australian National Insect Collection, CSIRO Ecosystem Sciences, Canberra, Australia

BPBM B.P. Bishop Museum, Hawaii, USA

MNHP Muséum National d’Histoire Naturelle, Paris, France

NHRS Swedish Museum of Natural History, Stockholm, Sweden

## Descriptions

### Acritoptila

Wells

http://species-id.net/wiki/Acritoptila

Acritoptila Wells (1982: 262); Kelley (1989: 190); Oláh and Johanson (2010a: 70).

#### Type species.

*Acritoptila globosa* Wells, 1982, by original designation.

#### Revised diagnosis.

Hydroptilinae with antennae comprising 26–41 flagellomeres in male and 24–26 flagellomeres in female; in male abdominal sternite VII bearing slender subapical spine mesally; abdominal segment VIII shorter than VII, broad; abdominal segment IX deeply excavated mid-ventrally, often produced distally as stout lateral lobes; in male genitalia, gonopods fused at least partially, not forming claspers, with paired, generally slender, elongate spines (“parameres”) laterally, arising from complex of internal apodemes, or with lateral margins of tergite X forming sclerotized spiny processes; phallic apparatus without titillator, often with complex spiny apical processes; in female, terminalia forming a short, broad oviscapt; final instar larvae laterally flattened, physogastric, head, thorax and first two abdominal segments slender, then abdominal segments increasing in size to fifth, decreasing distally from sixth, cuticle of head and thorax may have darkened bands or patches; case basically a laterally flattened purse of two equal valves, but shape and materials variable.

### Acritoptila disjuncta

Kelley

http://species-id.net/wiki/Acritoptila_disjuncta

**Figures 1–4.**
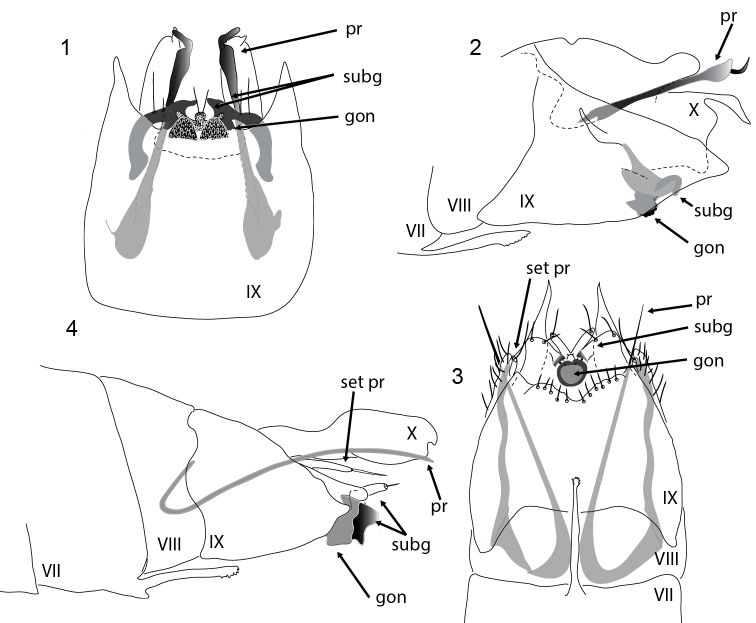
*Acritoptila* male genitalia. **1–2** *Acritoptila disjuncta* Kelley ventral and lateral views **3–4** *Acritoptila crinita* Kelley ventral and lateral view. Abbreviations: gon = gonopod(s); pr = parameres; set pr = setose process; subg = subgenital process(es); VII–X = abdominal segments VII–X.

Figs 1 , 2 , 24 , 25 , 30 , 31 , 35

Acritoptila disjuncta Kelley (1989: 193, figs 5, 6, 15, 16); Wells (1995: 235, figs 18, 19).

#### Revised diagnosis.

Males are recognised by genitalic features (Figs 1, 2): in ventral view by the conical gonopods with rugose surfaces, ventral to the sharply mesally directed darkly sclerotized subgenital processes with a small median papilla bearing a pair of setae and parameres that are dilated subapically proximal to a narrow constriction; females are readily distinguished by the mid ventral elongate digitiform process on abdominal segment VIII (Figs 24, 25). Males resemble most closely those of *Acritoptila chiasma* and *Acritoptila csavar* Oláh & Johanson, 2010a, all three species in lateral view having a pair of curved spines apically on tergite X. However, *Acritoptila chiasma* and *Acritoptila csavar* have paired sinuous elongate-slender parameres latero-ventrally, whereas in *Acritoptila disjuncta* these processes are constricted subapically and hooked apically; and *Acritoptila disjuncta* has well-developed apico-lateral lobes on abdominal segment IX.

**Figures 5–10.**
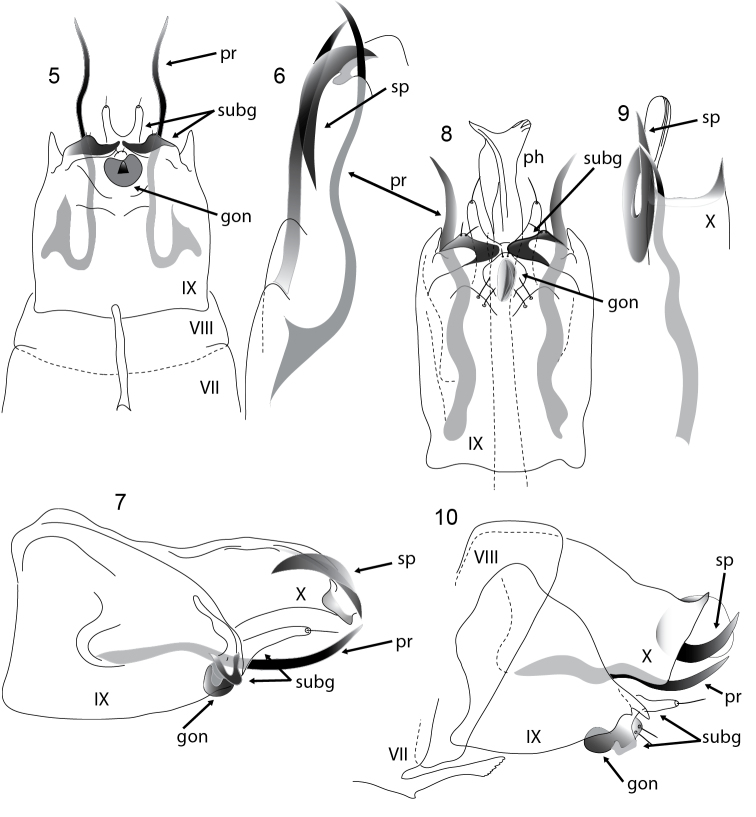
*Acritoptila* male genitalia. **5–7** *Acritoptila chiasma* Kelley ventral view, dorsal view of paramere and spines, and lateral view **8–10** *Acritoptila csavar* Oláh & Johanson ventral view, dorsal view of paramere and spines, and lateral view. Abbreviations: gon = gonopod(s); ph = phallic apparatus; pr = parameres; sp = spine on tergite X; subg = subgenital process(es); VII–X = abdominal segments VII–X.

Male antennae each with 30–34 flagellomeres; forewing length, 1.9–2.4 mm (n = 10).

Female antennae each with 24–26 flagellomeres; forewing length, 2.1–2.5 mm (n = 10).

#### Remarks.

*Acritoptila disjuncta* is widespread on the island (Fig. 35) and one of the most commonly collected of *Acritoptila* species at sites sampled in this study, although it was never as abundant in any collections as *Acritoptila crinita*. The larval cases, described and figured by Wells (1995), are basically rectangular secretion “purses” (Wells 1995: fig. 19). Many cases had a cover of sponge, always neatly shaped around the case, giving a spindle shape in profile (Fig. 30); it appears that the larva (Fig. 31) may crop the proliferating sponge.

#### Material examined.

Holotype male: New Caledonia, mountain stream up Boulari River, (BPBM); larvae, pupae, Province Sud, Ouenghi River nr Boulouparis, 20.xii.1983, A Wells, (ANIC); numerous males, females Province Sud, Dumbéa river, Branche sud, 22°08.344'S, 166°30.147'E, 42 m, 3.xi.2003, light trap, loc#006, KAJ (NHRS); numerous males, females, Province Sud, W part of Plaine des lacs, 150 m downstream bridge at La Capture, 22°15.967'S, 166°49.493'E, 261 m, 4–22.xi.2003, Malaise trap, loc#007, KAJ (NHRS); 2 females, Province Sud, Col d’Amieu, 319 m, small stony river, loc 23, 21°34.720'S, 165°49.620'E, Malaise trap, 30.xi–5.xii.2001, Johanson, Pape, Viklund (NHRS); 1 female, Province Sud, Col d’Amieu, 323 m, small stony river, loc 24, 21°34.844'S, 165°49.677'E, Malaise trap, 30.xi–5.xii.2001, Johanson, Pape, Viklund (NHRS); 1 female, Province Sud, Col d’Amieu, fauna reserve, 415 m, small forest stream, loc 25, 21°33.830'S, 165°45.584'E, Malaise trap, 30.xi–5.xii.2001, Johanson, Pape, Viklund (NHRS); 3 male, 7 females, Province Sud, stream draining to Marais de la Rivière Blanche, 1.35 km S Pont Pérignon, 22°08.496'S, 166°42.152'E, 180 m, 6–16.xi.2003, Malaise trap, loc#009, KAJ (NHRS); numerous males, females, Province Sud, stream draining to Marais de la Rivière Blanche, 2.25 km SW Pont Pérignon, 22.14158°’S, 166.67993 °E, 157 m, 6–16.xi.2003, Malaise trap, loc#010, KAJ (NHRS); 1 male, Province Sud, Monts Kwa Ne Mwa, on road between Noumea and Yaté, Rivière des Pirogues, 22°11.225'S, 166°43.338'E, 100 m, 7.xi.2003, light trap, loc#016, KAJ (NHRS); 1 male, Province Sud, Mt Dzumac, source stream of Ouinne River, downstream crosspoint to mountain track, 22°01.997'S, 166°28.486'E, 795 m, over about 30 m waterfall, 18.xi–4.xii.2003, Malaise trap, loc#031, KAJ (NHRS); numerous males, females, Province Sud, Tamoa River, 700m S road RT1 between Noumea and La Foa, 22°04.518'S, 166°16.592'E, 19.xi.2003, light trap, loc#033, KAJ (NHRS); numerous males, females, Province Sud, Hwa Hace Mtn, Hwa Motu River, at Pont Wamuttu, 1.0 km E Nassirah, about 200 m upstream bridge, 21°48.094'S, 166°04.298'E, 137 m, 20.xi–12.xii.2003, Malaise trap, loc#034, KAJ (NHRS); 1 male, 3 females, Province Sud, W slope Mt Ningua, Kwé Néco Stream, 3.9 km W summit of Mt Ningua, on Boulouparis-Thio Road, about 50 m upstream road, 21°44.359'S, 166°06.009'E, 117 m, 20.xi–12.xii.2003, Malaise trap, loc#035, KAJ (NHRS); 2 males, 18 females, Province Nord, Amoa River, 23 m, loc 20, 12 km W Poindimié, 22°58.092'S, 165°11.804'E, light trap, 26.xi.2001, Johanson, Pape, Viklund (NHRS); numerous males, females, Province Sud, Couvelée River at Haute Couvelée, 2.8 km SV summit of Mt Piditéré, 3.5 km NNE Dumbéa, 22°07.405'S, 166°28.023'E, 27 m, 28.xi.2003, light trap, loc#052, KAJ (NHRS); 6 males, 7 females, Province Sud, Xwé Pemöu Stream, 300 m N bridge over Dathio River at Atè, 6.2 km WNW Thio, 21.58835°S, 166.15117°E, 13 m, 29.xi.2003, light trap, loc#056, KAJ (NHRS); 1 male, Province Sud, lower part of Dumbéa River, 1.0 km SSW bridge over Dumbéa River at Dumbéa, 22°09.750'S, 166°26.700'E, 0.5 m, 30.xi.2003, light trap, loc#058, KAJ (NHRS); 1 male, numerous females, Province Sud, lower part Rivière des Pirogues, 800 m WNW summit of Mont Imbaah, 4.7 km E Lucky Creek in Plum, 22°18.559'S, 166°41.227'E, 1.3 m, 01.xii.2003, light trap, loc#059, KAJ (NHRS); 3 males, 6 females, Province Nord, 50 m upstream bridge on Hienghène-Tnèdo road, 3.9 km S summit of Mt Tnèda, 2.2 km E Tnèdo, 20°43.085'S, 164°49.928'E, 29 m, 7.xii.2003, light trap, loc#071, KAJ (NHRS); numerous males, females, Province Nord, Wé Caot Stream, draining NNE side of Mt Panié, 0.9 km NW Cascade de Tao, 20°33.311'S, 164°48.064'E, 18.xii.2003, light trap, loc#084, KAJ (NHRS); 1 female, Province Nord, Wan Pwé On Stream, draining NNE side of Mt Panié, 3.9 km NW Cascade de Tao, 20°31.820'S, 164°47.016'E, 18.xii.2003, light trap, loc#085, KAJ (NHRS); numerous males, females, Province Nord, Bouérabate Stream, S Mont Ninndo, along road Barabache-Boulagoma, 20°17.409'S, 164°11.242'E, 60 m, 19.xii.2003–7.i.2004, Malaise trap, loc#089, KAJ (NHRS); numerous males, females, Province Nord, Rivière Néhoué, camp Amenage de Néhoué, 20°25.037'S, 164°13.222'E, 12 m, 19.xii.2003, light trap, loc#090, KAJ (NHRS); numerous males, females, Province Nord, Héémwâ Pwei River, 50 m upstream bridge on Touho-Hienghène road, 1.0 km N Paola, 20.76512°S, 165.10979°E, 22.xii.2003, light trap, loc#095, KAJ (NHRS); numerous males, females, Province Nord, Ponandou Tiôgé River at Kögi, 3.9 km SSW Touho, 20°49.043'S, 165°13.551'E, 25 m, 26.xii.2003, light trap, loc#100, KAJ (NHRS); 1 male, numerous females, Province Sud, W slope Mt Ningua, Kwé Néco Stream, at Camp Jacob, 3.9 km W summit of Mt Ningua, on Boulouparis-Thio Road, about 50 m upstream road, 21°44.083'S, 166°06.298'E, 117 m, 29.xi.2003–12.xii.2003, Malaise trap, loc#053, KAJ (NHRS); 4 males, numerous females, New Caledonia, Province Nord, Plaine des Gaïacs, Rivière Rouge, 14.2 km NW summit of Mt Rouge, 50 m upstream road RT1 Noumea-Koné, 20°31.573'S, 164°46.690'E, 23 m, 2.i.2004, light trap, loc#104, KAJ (NHRS); 1 female, New Caledonia, province Sud, Kuébini River (Kwé Binyi River), 1.4 km N summit of Mt Nokowèito, inland Baie de Tere, 13.5 km SSW Yaté, 22°15.467'S, 167°00.238'E, 1 m, 6.i.2004, light trap, loc#111, KAJ (NHRS); numerous males, females, New Caledonia, Province Nord, 2.8 km ENE Bopope, Rivière Oua Mendiou, 100 m S RPN2 Koné-Poindimié, 20°54.455'S, 165°06.300'E, 78 m, 14.i.2004, light trap, loc#119, KAJ (NHRS).

### Acritoptila crinita

Kelley

http://species-id.net/wiki/Acritoptila_crinita

Figs 3 , 4 , 26 , 32 , 33 , 35

Acritoptila crinita Kelley (1989: 193, figs 4, 13, 14).
Acritoptila karika Oláh & Johanson (2010a: 70), syn. n.

#### Revised diagnosis.

The males of this species are most closely similar to *Acritoptila chiasma* and *Acritoptila csavar* with which it shares the strongly reduced, fused form of the gonopods, and the slender, elongate ventro-lateral processes or parameres; but they can distinguished because in *Acritoptila crinita* the parameres are only slightly curved, not sinuous as in the other two species (Figs 3, 4). In addition, *Acritoptila crinita* lacks the sharp apico-lateral spines seen on tergite X of *Acritoptila chiasma* and *Acritoptila csavar* and *Acritoptila crinita* has a pair of lateral digitiform apically setose processes on tergite X. Females are recognised by the very dark apices of the paired lobes of sternite IX. Larval and pupal cases are rectangular purses (Fig. 32), obliquely sloped at each end, constructed of secretion with diatoms accreted smoothly into walls.

Male antennae each with 29–33 flagellomeres; forewing length, 1.5–2.0 mm (n = 10).

Female antennae each with 23 flagellomeres; forewing length, 2.8–2.1 mm (n = 10).

#### Remarks.

Of all species of *Acritoptila*, *Acritoptila crinita* was collected most commonly by Johanson and colleagues in New Caledonia, often being taken in large numbers at sites in both the north and south. The males are readily recognised in ventral view by the fused, darkly sclerotized, rounded to heart-shaped ventral genitalic structure interpreted as the fused gonopods.

The features by which Oláh and Johanson (2010a) distinguished *Acritoptila karika* Oláh & Johanson, 2010a from *Acritoptila crinita* are “… segment X without sclerotized apical structures; fused ring-shaped gonopods without dorsal projection; basal plate with short digitiform processes; and apex of the phallic organ with a lobe-like complex (not with spine-like structures)”; *Acritoptila karika* has “Segment X … slightly sclerotized horizontally…”. This is simply another interpretation of the sclerotization displayed in the type of *Acritoptila crinita*. *Acritoptila crinita* also has fused gonopods without a dorsal projection, but has the basal plate (= bilobed processes of Kelley (1989)) with short digitiform processes as in *Acritoptila karika*; and the phallic organ has the same apical features that can be interpreted as spiny or lobe-like. Hence we are synonymising *Acritoptila karika* with *Acritoptila crinita*. In fact, numerous male and female specimens identified as *Acritoptila crinita* were collected from the type locality of *Acritoptila karika*, and also at a site from which 2 paratypes were designated.

#### Material examined.

Holotype male: *Acritoptila crinita* Kelley, New Caledonia, headwaters of Honailu River (BPBM); Holotype male: *Acritoptila karika* Oláh & Johanson, New Caledonia, Province Nord, 50 m upstream bridge on Hienghène-Tnèdo road, 3.9 km S summit of Mt Tnèdo, 2.2 km E Tnèdo, 20°43.085'S, 164°49.928'E, loc#071 (MNHN); immatures, Province Nord, Bopope, 18.xii.1983, A Wells, (ANIC); numerous males and females, Province Nord, Amoa River, 23 m, loc 20, 12 km W Poindimié, 22°58.092'S, 165°11.804'E, light trap, 26.xi.2001, Johanson, Pape, Viklund (NHRS); 12 males, Province Sud, Monts Kwa Ne Mwa, on road between Noumea and Yaté, 2.0 km E Pic Mouirange, 22°12.356'S, 166°40.798'E, 220 m, 7–16.xi.2003, Malaise trap, loc#014, KAJ (NHRS); 1 male, Province Sud, Mt Dzumac, source stream of Ouinne River, near crosspoint to mountain track, 22°02.439'S, 166°28.646'E, 805 m, 18.xi–4.xii.2003, Malaise trap, loc#029, KAJ (NHRS); numerous males, females, Province Sud, Couvelée River at Haute Couvelée, 2.8 km SV summit of Mt Piditéré, 3.5 km NNE Dumbéa, 22°07.405'S, 166°28.023'E, 27 m, 28.xi.2003, light trap, loc#052, KAJ (NHRS); numerous males, females, Province Sud, Xwé Pemöu Stream, 300 m N bridge over Dathio River at Atè, 6.2 km WNW Thio, 21.58835°S, 166.15117°E, 13 m, 29.xi.2003, light trap, loc#056, KAJ (NHRS); 3 males, Province Sud, lower part of Dumbéa River, 1.0 km SSW bridge over Dumbéa River at Dumbéa, 22°09.750'S, 166°26.700'E, 0.5 m, 30.xi.2003, light trap, loc#058, KAJ (NHRS); numerous males, females, Province Nord, Wemwâdiu stream, 850 m E summit Kögi Mtn, 5 m upstream road, about 200 m S Tiwaka River, 20°49.020'S, 165°14.165'E, 24 m, 6–27.xii.2003, Malaise trap, loc#067, KAJ (NHRS); numerous males, Province Nord, 50 m upstream bridge on Hienghène-Tnèdo road, 3.9 km S summit of Mt Tnèda, 2.2 km E Tnèdo, 20°43.085'S, 164°49.928'E, 29 m, 7.xii.2003, light trap, loc#071, KAJ (NHRS); numerous males, females, Province Nord, 1 m upstream road, below waterfall on Hienghène-Tnèdo road, 2.2 km SSW summit of Mt Unpac, 4.9 km ESE Tnèdo, 20.73879°S, 164.85508°E, 7.xii.2003, light trap, loc#072, KAJ (NHRS); numerous males, females, Province Nord, 2.8 km ENE Bopope, Rivière Oua Mendiou, 100 m S RPN2 Koné-Poindimié, 20°54.455'S, 165°06.300'E, 78 m, 14.xii.2003, light trap, loc#119, KAJ (NHRS); 3 males (1 dissected by KAJ as B4), Province Nord, Wan Pwé On Stream, draining NNE side of Mt Panié, 3.9 km NW Cascade de Tao, 20°31.820'S, 164°47.016'E, 18.xii.2003, light trap, loc#085, KAJ (NHRS); 3 males, Province Nord, Bouérabate Stream, S Mont Ninndo, along road Barabache-Boulagoma, 20°17.409'S, 164°11.242'E, 60 m, 19.xii.2003–7.i.2004, Malaise trap, loc#089, KAJ (NHRS); numerous males, females, Province Nord, Rivière Néhoué, camp Amenage de Néhoué, 20°25.015'S, 164°13.245'E, 12 m, 19.xii.2003, light trap, loc#091, KAJ (NHRS); numerous males, females, Province Nord, Ponandou Tiôgé River at Kögi, 3.9 km SSW Touho, 20°49.043'S, 165°13.551'E, 25 m, 26.xii.2003, light trap, loc#100, KAJ (NHRS); numerous males, females, Province Nord, Plaine des Gaïacs, Rivière Rouge, 14.2 km NW summit of Mt Rouge, 50 m upstream road RT1 Noumea-Koné, 20°31.573'S, 164°46.690'E, 23 m, 2.i.2004, light trap, loc#104, KAJ (NHRS); 3 males, 2 females, Province Nord, Établissement thermal de la Crouen, along Riv. la Crouen, 30 m upstream road RM3, 21°32.105'S, 165°53.319'E, 15 m, 5.i.2004, Malaise trap, loc#110, KAJ (NHRS); males (1 dissected by KAJ as ‘Y’), Province Sud, Col d’Amieu, Xwé Ko River, on road to St. Forestière, 21°35.612'S, 165°48.241'E, 368 m, 8.i.2004, light trap, loc#114, KAJ (NHRS); 4 males, 3 females, Province Sud, Sarraméa, Xwê Wya River, 21°38.318'S, 165°51.582'E, 127 m, 17–18.i.2004, light trap, loc#121, KAJ (NHRS).

### Acritoptila chiasma

Kelley

http://species-id.net/wiki/Acritoptila_chiasma

Figs 5–7 , 27 , 35

Acritoptila chiasma Kelley (1989: 192, figs 2, 11, 12).

#### Revised diagnosis.

Males of *Acritoptila chiasma* are similar to *Acritoptila crinita* and *Acritoptila csavar*, with which they share, in ventral view, the rather similar tongue-shaped form of the mid ventral genitalic structures interpreted as subgenital processes (Fig. 5). The males are distinguished from *Acritoptila crinita* by having spiny apical processes apico-laterally on tergite X (Fig. 6), which are hooked in *Acritoptila csavar* (Fig. 9), and simply curved in *Acritoptila chiasma* (Figs 6, 7). Neither *Acritoptila chiasma* nor *Acritoptila csavar* has the lateral setose processes seen on tergite X of *Acritoptila crinita*.

Male antennae each with 37–40 flagellomeres; forewing length, 2.0–2.1 mm (n = 3).

#### Material examined.

Holotype male: New Caledonia, mountain stream up Boulari River, (BPBM); 3 males (2 on slides), 4 females (1 on slide), Province Sud, lower part Rivière des Pirogues, 800 m WNW summit of Mont Imbaah, 4.7 km E Lucky Creek in Plum, 22°18.559'S, 166°41.227'E, 1.3 m, 1.xii.2003, light trap, loc#059, KAJ (NHRS); 1 male, Province Sud, Mt Dzumac, source stream of Ouinne River, at crosspoint to mountain track, 22°02.218'S, 166°28.566'E, 797 m, 18.xi.2003, light trap, loc#032, KAJ (NHRS).

#### Remarks.

The features separating *Acritoptila chiasma* from *Acritoptila csavar* are weak, but appear to be definitive. In the diagnosis of *Acritoptila chiasma*, Kelley (1989) states that species “is most closely related to *Acritoptila crinita*”, but has the tenth tergum “quite distinctive”. However, *Acritoptila chiasma* more closely resembles *Acritoptila csavar*, both having gonopods of similar shape and stout spiny processes laterally on tergite X, whereas *Acritoptila crinita* has the gonopods forming a tight sphere and on tergite X has slightly sclerotized, weakly curved, lateral processes. *Acritoptila chiasma* differs from *Acritoptila csavar* in having in ventral the structure representing the fused gonopods more rounded, and in dorsal view the apical angles of tergite X acute and in lateral view the spine on tergite X curved ventrad, rather than dorsad. This species has been collected only in the far south of the island.

### Acritoptila csavar

Oláh & Johanson

http://species-id.net/wiki/Acritoptila_csavar

Figs 8–10

Acritoptila csavar Oláh & Johanson (2010a: 70, figs 1–3).

#### Revised diagnosis.

Males of *Acritoptila csavar* most closely resemble those of *Acritoptila chiasma*, from which it they are distinguished by the presence of hooked (Fig. 9) rather than gently curving (Fig. 6) apico-lateral spines on tergite X (also see diagnoses for *Acritoptila crinita* and *Acritoptila chiasma*), and in ventral view by the paler and more ovoid shape of the fused gonopods.

Male antennae each with 39–40 flagellomeres; forewing length, 2.0–2.3 mm (n = 6).

#### Material examined.

Paratype male, Province Sud, Tamoa River, 700 m S road RT1 between Noumea and La Foa, 22°04.518'S, 166°16.592'E, 19.xi.2003, light trap, loc#033, KAJ (NHRS); 5 males (2 on slides), Province Nord, Ponandou Tiôgé River at Kögi, 3.9 km SSW Touho, 20°49.043'S, 165°13.551'E, 25 m, 26.xii.2003, light trap, loc#100, KAJ (NHRS); 3 males (KAJ sp ‘G’), New Caledonia, Province Nord, Forêt Plate, Ouendé River, at 2.5 km WNW summit of Katépouenda, 23.3 km E Pouembout, 21°07.490'S, 165°06.723'E, 470 m, 8–15.i.2004, Malaise trap, loc#112, KAJ (NHRS).

#### Remarks.

Few specimens of this species have been collected, several from the north and several from the south (Fig. 35). See also Remarks under *Acritoptila chiasma*.

### Acritoptila glossocercus

Kelley

http://species-id.net/wiki/Acritoptila_glossocercus

Figs 11 , 12

Acritoptila glossocercus Kelley (1989: 193, figs 7, 17, 18).

#### Diagnosis.

**Figures 11–16.**
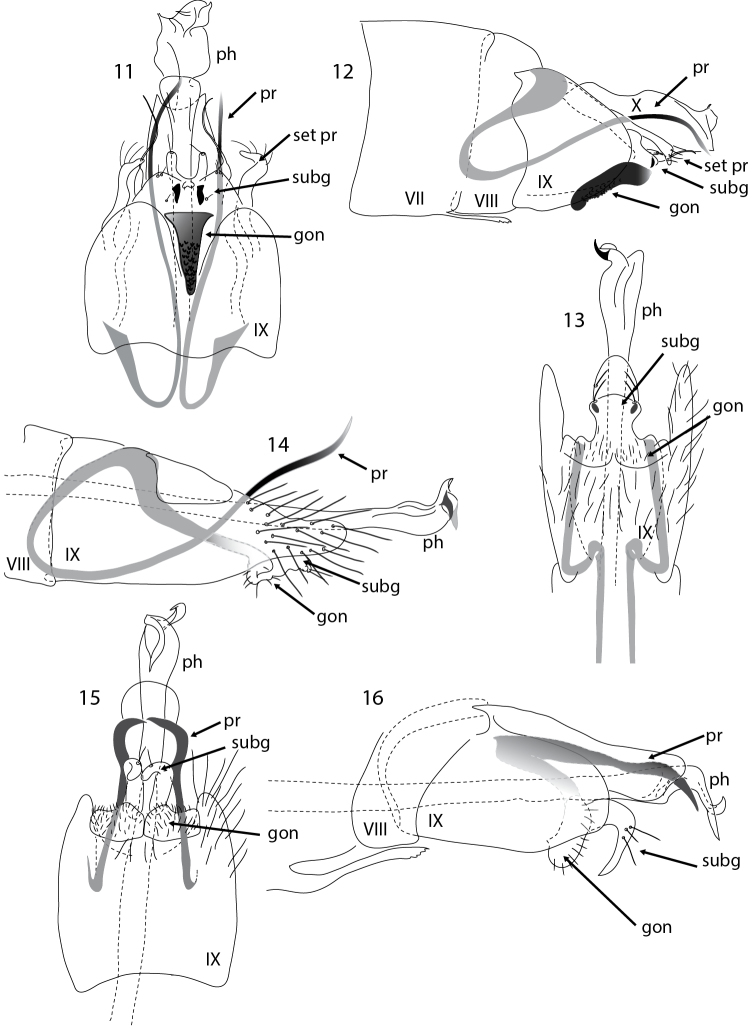
*Acritoptila* male genitalia. **11–12** *Acritoptila glossocercus* Kelley ventral and lateral views **13–14** *Acritoptila parallela* sp. n. ventral and lateral views **15–16** *Acritoptila amphapsis* Kelley ventral and lateral views. Abbreviations: gon = gonopod(s); ph = phallic apparatus; pr = parameres; set pr = setose process; subg = subgenital process(es); VII-X = abdominal segment VII–X.

This species is distinctive in the genus in having a single mid-ventral very darkly sclerotized tongue-like process, interpreted as the fused gonopods. It groups with *Acritoptila crinita*, *Acritoptila chiasma* and *Acritoptila csavar* in having filamentous parameres, but particularly with *Acritoptila crinita* in having paired setose processes laterally on tergite X (Figs 11, 12).

#### Revised description.

Male antennae each with 29–32 flagellomeres, with large sensilla placodea on surfaces; forewing length, 1.9–2.3 mm (n = 7). Male genitalia (Figs 11, 12). Abdominal segment VII bearing a slender elongate process mid-ventrally. Abdominal segment VIII much shorter than IX, which is excavated mid-ventrally, accommodating darkly sclerotized, rugose tongue-like process interpreted as fused gonopods. Abdominal segment X broad based, concave apically, with two small spines medially, and laterally an elongate apically setose process. Subgenital processes, in ventral view, in form of small conical lobes, each bearing a robust seta meso-ventrally; dorsally a pair of membranous setose lobes. Paired thread-like straight parameres extend distally from robust apodemes arising from base of segment IX. Phallic apparatus stout, constricted sub-apically, a strap-like band at apex. Female unknown.

#### Remarks.

**Figures 17–21.**
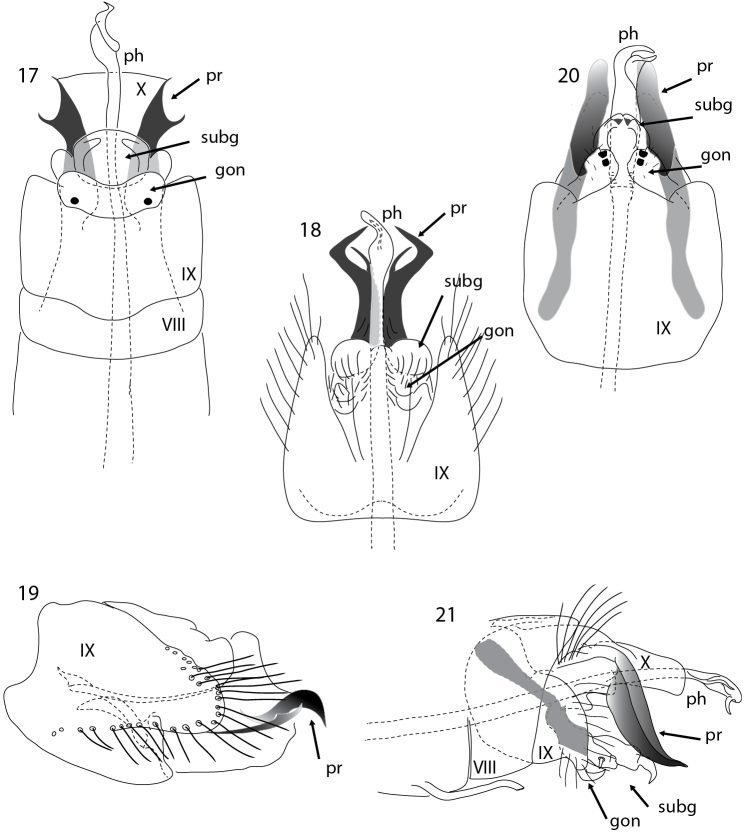
*Acritoptila* male genitalia. **17** *Acritoptila planichela* Kelley ventral view **18–19** *Acritoptila forficata* sp. n. ventral and lateral views **20** *Acritoptila ouenghica* Wells ventral view **21** *Acritoptila macrospina* sp. n. lateral view. Abbreviations: gon = gonopod (s); ph = phallic apparatus; pr = parameres; subg = subgenital process(es); VIII-X = abdominal segments VIII-X.

Upon examination, the holotype male was found to be identical in all respects with a group of specimens collected from the sites listed below, save in the form of the mid-ventral structure illustrated and described by Kelley (1989: 193) as “tongue-shaped in caudal view”, yet shown as a small rounded structure in his figure of ventral view (Fig. 17, Fig. 11). In fact, in the type this structure has been broken off (from Kelley’s fig. 7, it appears it may have been intact when he drew his lateral view). The few known specimens of *Acritoptila glossocercus* were all collected in northern New Caledonia (Fig. 35).

#### Material examined.

Holotype male: New Caledonia, mountain stream up Boulari River, (BPBM); 1 male, Province Sud, Monts Kwa Ne Mwa, on road between Noumea and Yaté, Rivière des Pirogues, 22°11.225'S, 166°43.338'E, 100 m, 7.xi.2003, light trap, loc#016, KAJ (NHRS); 9 males (3 on slides), Province Nord, 50 m upstream bridge on Hienghène-Tnèdo road, 3.9 km S summit of Mt Tnèda, 2.2 km E Tnèdo, 20°43.085'S, 164°49.928'E, 29 m, 7.xii.2003, light trap, loc#071, KAJ (NHRS); 3 males, New Caledonia, Province Nord, Ponandou Tiôgé River at Kögi, 3.9 km SSW Touho, 20°49.043'S, 165°13.551'E, 25 m, 26.xii.2003, light trap, loc#100, KAJ (NHRS); 1 male (on slide), Province Nord, Bouérabate Stream, S Mont Ninndo, along road Barabache-Boulagoma, 20°17.409'S, 164°11.242'E, 60 m, 19.xii.2003–7.i.2004, Malaise trap, loc#089, KAJ (NHRS).

### Acritoptila parallela sp. n.

http://zoobank.org/F31DAC91-2622-4DB5-AE43-C429A7D49EA6

http://species-id.net/wiki/Acritoptila_parallela

Figs 13 , 14 , 29 , 35

#### Diagnosis.

This species resembles *Acritoptila glossocercus*, *Acritoptila chiasma*, *Acritoptila csavar* and *Acritoptila crinita* in having elongate ventro-lateral spiny parameres, but differs in that apico-laterally abdominal segment VIII is produced to form pronounced lateral lobes that extend distally beyond the apices of gonopods, and gonopods and subgenital processes appear in ventral view to form a single broad-base, medially constricted plate. The females have a small elongate anchor-shaped marking ventrally on abdominal segment IX.

#### Description, male.

Male antennae each with 27–29 flagellomeres, bicoloured, apical 4 segments pale, more proximally 11 dark, rest pale; forewing length 2.0–2.2 mm (n = 6). Female antennae each with 24 flagellomeres; forewing length 2.1–2.2 mm (n = 2). Male genitalia (Figs 13, 14). Abdominal segment VII bearing slender elongate spine midventrally. Abdominal segment IX produced posteriorly, forming parallel-sided lobes, in lateral view segment narrows abruptly towards rounded apices. Gonopods and subgenital processes in ventral view appear to be fused to form a plate, broad at base, constricted medially, bearing a pair of dark knob-like setae at apico-lateral angles. Paired thread-like almost straight parameres extend distally from robust apodemes arising at base of segment IX. Phallic apparatus narrow, dilated towards apex, a sharp, sclerotized spur at right angles apically. Female genitalia (Fig. 29). Abdominal segment IX in ventral view with a pair of lobes laterally and median anchor-shaped gland.

#### Material examined.

**Holotype:** male, New Caledonia, Province Nord, Mt Panié, stream at camp, 20.58139°S, 164.76444°E, 1310 m, 9.xii.2003–2.i.2004, Malaise trap, loc#074, KAJ, (MNHP); **paratypes:** 12 males (2 on slides), 12 females (2 on slides), same data as for holotype (NHRS).

#### Etymology.

*parallela*, named for the nearly parallel arrangement of several structures in the male genitalia.

#### Remarks.

*Acritoptila parallela* is known only from the type locality in the northeast of the island.

### Acritoptila amphapsis

Kelley

http://species-id.net/wiki/Acritoptila_amphapsis

Figs 15 , 16 , 28 , 34 , 35

Acritoptila amphapsis Kelley (1989: 191, figs 1, 9, 10); Wells (1995: 238, fig. 17).

#### Revised diagnosis.

Males of *Acritoptila amphapsis* are distinctive, being distinguished from males of other *Acritoptila* species by their genitalia in ventral view with parameres in form of pair of mesally directed, horn-like spines postero-lateral to gonopods (Fig. 15) and, in lateral view, coarsely hooked apices of the “ventral processes” (as termed by Kelley 1989), here interpreted as subgenital processes (Figs 15, 16). Females are distinguished by the apico-mesal concavity and sclerotised plate-like gland on sternite VIII (Fig. 28). Male antennae each with 31–35 flagellomeres, bicoloured with distal dark band of 9 flagellomeres followed by 9 pale flagellomeres apically; forewing length, 1.9–2.0 mm (n = 4). Female antennae each with 24–26 flagellomeres, bicoloured with distal dark band of 6 segments followed by 6 pale apically; forewing length 1.8–2.0 mm (n = 5).

#### Remarks.

This species was not commonly collected, but was taken in both the northern and southern provinces (Fig. 35). It was identified only in samples taken in the wet season, from late November, with the largest sample dated 8–15 January. This could indicate a restricted period of emergence, or possibly a later time of emergence than for other congeners. A pupal case attributed to this species by Wells (1995: fig. 17) and pictured here in Fig. 34 is a subrectangular purse case constructed of fine sand grains.

#### Material examined.

Holotype male: New Caledonia, Honailu River, (BPBM); cases, cased pupa, Province Sud, creek between Negropa and Koh on La Foa-Canala Road, 19 Dec. 1983, A Wells (ANIC); 1 male (on slide), Province Sud, W slope Mt Ningua, Kwé Néco, Stream, at Camp Jacob, 3.7 km WNW summit of Mt Ningua, on Boulouparis-Thio Road, about 50 m upstream road, 21°43.613'S, 166°06.567'E, 150 m, 29.xi–12.xii.2003, Malaise trap, loc#054, KAJ (NHRS); 1 male, Province Nord, 50 m upstream bridge on Hienghène-Tnèdo road, 3.9 km S summit of Mt Tnèda, 2.2 km E Tnèdo, 20°43.085'S, 164°49.928'E, 29 m, 7.xii.2003, light trap, loc#071, KAJ (NHRS); 23 males, 54 females (2 males, 2 females on slides), Province Nord, Bouérabate Stream, S Mont Ninndo, along road Barabache-Boulagoma, 20°17.409'S, 164°11.242'E, 60 m, 19.xii.2003–7.i.2004, Malaise trap, loc#089, KAJ (NHRS); 1 male Province Nord, Forêt Plate, Ouendé River, at 2.5 km WNW summit of Katépouenda, 23.3 km E Pouembout, 21°07.490'S, 165°06.723'E, 470 m, 8–15.i.2004, Malaise trap, loc#112, KAJ (NHRS).

### Acritoptila planichela

Kelley

http://species-id.net/wiki/Acritoptila_planichela

Fig. 17

Acritoptila planichela Kelley (1989: 194).

#### Revised diagnosis.

In having the parameres branched, *Acritoptila planichela* resembles *Acritoptila forficata*, sp. n. however in *Acritoptila forficata* the parameres are more slender and the mesal branch is the shorter, finer branch and closely associated with the lateral branch whereas in *Acritoptila planichela* the lateral branch is shorter and finer than the lateral branch, and *Acritoptila planichela* lacks the pronounced lateral lobes on abdominal segment IX seen in *Acritoptila forficata* sp. n. *Acritoptila planichela* shares with *Acritoptila ouenghica* and *Acritoptila macrospina* sp. n. the feature of curiously modified knob-like setae on the fused, non-sclerotized gonopods, but neither of those species has branched parameres. Male antennae damaged in only specimens at hand; forewing length, 2.1 mm (n = 1).

#### Remarks.

Only a single specimen was collected despite the extensive field work. Thus, with the 3 identified by Kelley (1989), 4 specimens are now known, all from the southern province.

#### Material examined.

Holotype male: New Caledonia, mountain stream up Boulari River, (BPBM); 1 male (on slide), Province Sud, Monts Kwa Ne Mwa, on road between Noumea and Yaté, 1.5 km E Pic Mouirange, 22°12.545'S, 166°40.246'E, 143 m, 9.xi.2003, light trap, loc#018, KAJ (NHRS).

### Acritoptila forficata sp. n.

http://zoobank.org/606C0AF2-B93C-4B6B-8988-E659480FD608

http://species-id.net/wiki/Acritoptila_forficata

Figs 18 , 19 , 35

#### Diagnosis.

Superficially, males of *Acritoptila forficata* resemble those of *Acritoptila parallela* sp. n., having similar elongate lateral lobes on abdominal segment IX. However, *Acritoptila forficata* has prominent forked parameres, rather than the fine filaments that characterize *Acritoptila parallela* and in that feature resemble *Acritoptila planichela*, although the parameres are more slender, and their forks more pronounced; *Acritoptila planichela* lacks lateral lobes on abdominal segment IX.

#### Description.

Male antennae each with 29–31 flagellomeres, with large *sensilla placodea* on surfaces; forewing length 2.1–2.3 mm (n = 3). Male genitalia (Figs 18, 19). Abdominal segment VII bearing a slender elongate process mid-ventrally. Abdominal segment IX in ventral view laterally produced posteriorly to form prominent lateral lobes. Gonopods and subgenital processes appear to be fused to form a rounded structure, apico-laterally rounded. Paired forked spiny parameres arise from stout apodemes. Phallic apparatus slender medially, dilated towards apex, a strap-like twist apically. Female unknown.

#### Material examined.

Holotype male (on slide): New Caledonia, Province Sud, Monts des Koghis, ca 800 m S Koghi Restaurant, 22.18406°S, 166.50383°E, 420 m, 11–26.xi.2003, Malaise trap, loc#022, KAJ (MNHP); **Paratypes:** 2 males, Province Nord, Mt Panié, stream at camp, 20.58139°S, 164.76444°E, 1310 m, 9.xii.2003–2.i.2004, Malaise trap, loc#074, KAJ (NHRS).

#### Etymology.

Named for the forked appearance of the parameres.

#### Remarks.

Only 3 specimens of this species are known, from two widely separated localities, one in the south, the other in the north.

### Acritoptila ouenghica

Wells

http://species-id.net/wiki/Acritoptila_ouenghica

Fig. 20

Acritoptila ouenghica Wells (1995: 235).

#### Revised diagnosis.

*Acritoptila ouenghica* groups with *Acritoptila planichela* and *Acritoptila macrospina* sp. n. in having, in the male genitalia, curiously modified setae on the fused gonopods, described as “tab-like” by Wells (1995) but in the two other species more knob-like. Unlike other New Caledonian congeners, *Acritoptila ouenghica* lacks the free parameres, the parameres instead appear to be fused laterally as broad sclerotized margins on tergite X, although in cleared specimens these clearly arise from stout apodemes. Male antennae each with 30–33 flagellomeres; forewing length 1.9–2.2 mm (n = 8).

#### Remarks.

Very few specimens of *Acritoptila ouenghica* were taken in all the recent collecting — one specimen from the south and several from the north (Fig. 35) — and no females have been associated.

#### Material examined.

Holotype male: New Caledonia, Ouenghi River, nr Boulouparis (ANIC); 1 male, Province Sud, Couvelée River at Haute Couvelée, 2.8 km SV summit of Mt Piditéré, 3.5 km NNE Dumbéa, 22°07.405'S, 166°28.023'E, 27 m, 28.xi.2003, light trap, loc#052, KAJ (NHRS); 5 males Province Nord, 50 m upstream bridge on Hienghène-Tnèdo road, 3.9 km S summit of Mt Tnèda, 2.2 km E Tnèdo, 20°43.085'S, 164°49.928'E, 29 m, 7.xii.2003, light trap, loc#071, KAJ (NHRS); 4 males. Province Nord, Ponandou Tiôgé River at Kögi, 3.9 km SSW Touho, 20°49.043'S, 165°13.551'E, 25 m, 26.xii.2003, light trap, loc#100, KAJ (NHRS).

### Acritoptila macrospina sp. n.

http://zoobank.org/FD1907D2-8250-4909-A886-027FEFA5F496

http://species-id.net/wiki/Acritoptila_macrospina

Figs 21 –23 , 35

#### Diagnosis.

The males of this species differ from all other New Caledonian species in having among genitalic structures stout, sclerotized asymmetrical parameres, in ventral view sharply angled mesally.

#### Description, male.

Antennae each with 26–31 flagellomeres, with large *sensilla placodea* on surfaces; forewing length 1.9–2.0 mm (n = 5).

**Figures 22–23.**
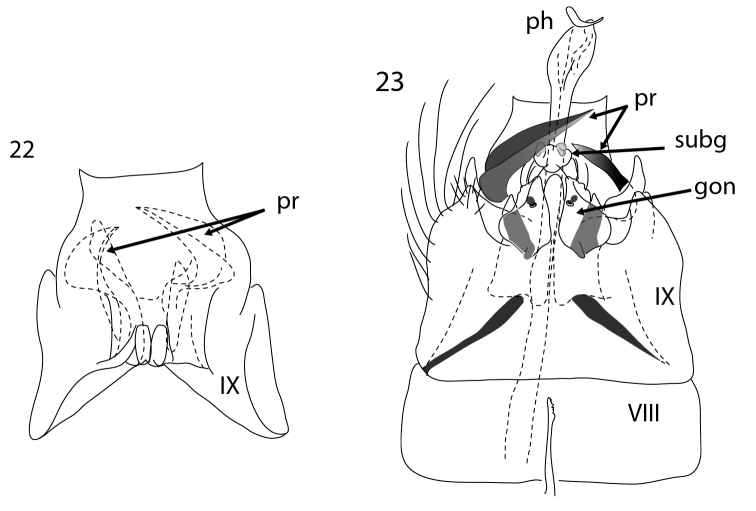
*Acritoptila macrospina* sp. n. lateral and dorsal ventral views. Abbreviations: gon = gonopod(s); ph = phallic apparatus; pr = parameres; subg = subgenital process(es); VIII–IX = abdominal segments VIII–IX.

Male genitalia (Figs 21–23). Abdominal segment VII bearing a slender elongate process mid-ventrally. Abdominal segment VIII shorter than IX. Abdominal segment IX in lateral view broader than long, in ventral view widely excavated apico-mesally. Gonopods in ventral view in form of discrete triangular lobes, each with a small rounded knob-like seta at about 2/3 length. Subgenital processes irregular in shape, in ventral view forming rounded lobe medially and pair of apically acute lobes laterally. Parameres leaf-shaped, left longer than right, in lateral view sharply down-turned, in ventral view directed mesad. Phallic apparatus elongate, dilated subapically with a slender re-curved apical spine. Female: unknown.

#### Material examined.

Holotype male: New Caledonia, 1 male (on slide), Province Nord, Wemwâdiu stream, 850 m E summit Kögi Mtn, 5 m upstream road, about 200 m S Tiwaka River, 20°49.020'S, 165°14.165'E, 24 m, 6–27.xii.2003, Malaise trap, loc#067, KAJ (MNHP). **Paratypes:** 33 males (3 on slides), Province Nord, Ponandou Tiôgé River at Kögi, 3.9 km SSW Touho, 20°49.043'S, 165°13.551'E, 25 m, 26.xii.2003, light trap, loc#100, KAJ (NHRS); 1 male, Province Nord, Plaine des Gaïacs, Rivière Rouge, 14.2 km NW summit of Mt Rouge, 50 m upstream road RT1 Noumea-Koné, 20°31.573'S, 164°46.690'E, 23 m, 2.i.2004, light trap, loc#104, KAJ (NHRS).

#### Etymology.

Named for the stout spines in the male genitalia.

#### Remarks.

Collected from only 3 northern localities (Fig. 35).

**Figures 24–29.**
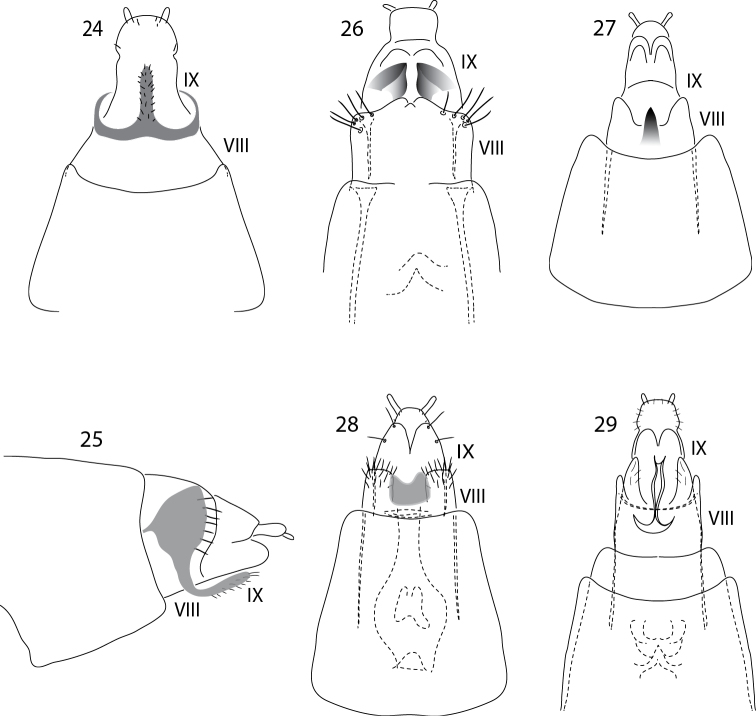
*Acritoptila* female terminalia. **24–25** *Acritoptila disjuncta* Kelley ventral and lateral views **26** *Acritoptila crinita* ventral view **27** *Acritoptila chiasma* Kelley ventral view **28** *Acritoptila amphapsis* Kelley ventral view **29** *Acritoptila parallela* sp. n. ventral view. Abbreviations: VIII–IX = abdominal segments VIII–IX.

**Figures 30–34.**
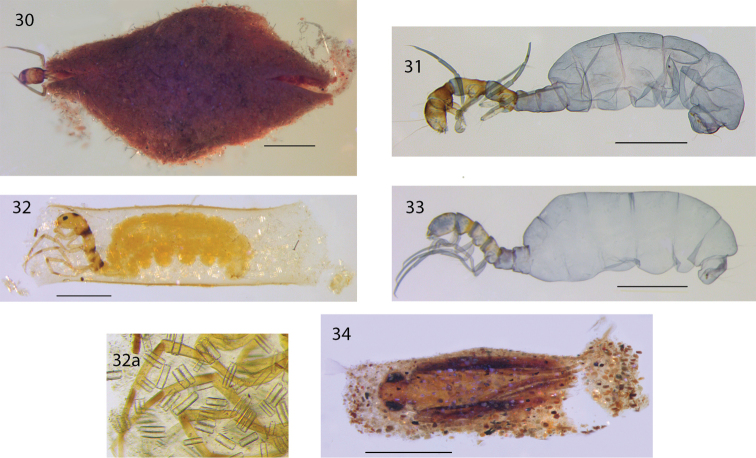
*Acritoptila* larvae and cases. **30–31** *Acritoptila disjuncta* Kelley larva in sponge-covered case and larva removed from case, lateral view **32–33** *Acritoptila crinita* Kelley larva in case of secretion and (32a) embedded diatoms and larva removed from case, lateral view **34** *Acritoptila amphapsis* Kelley pupa in damaged sand grain case. Scale bars: **30, 32** = 0.5 mm; **31, 33, 34** = 1.0 mm.

**Figure 35.**
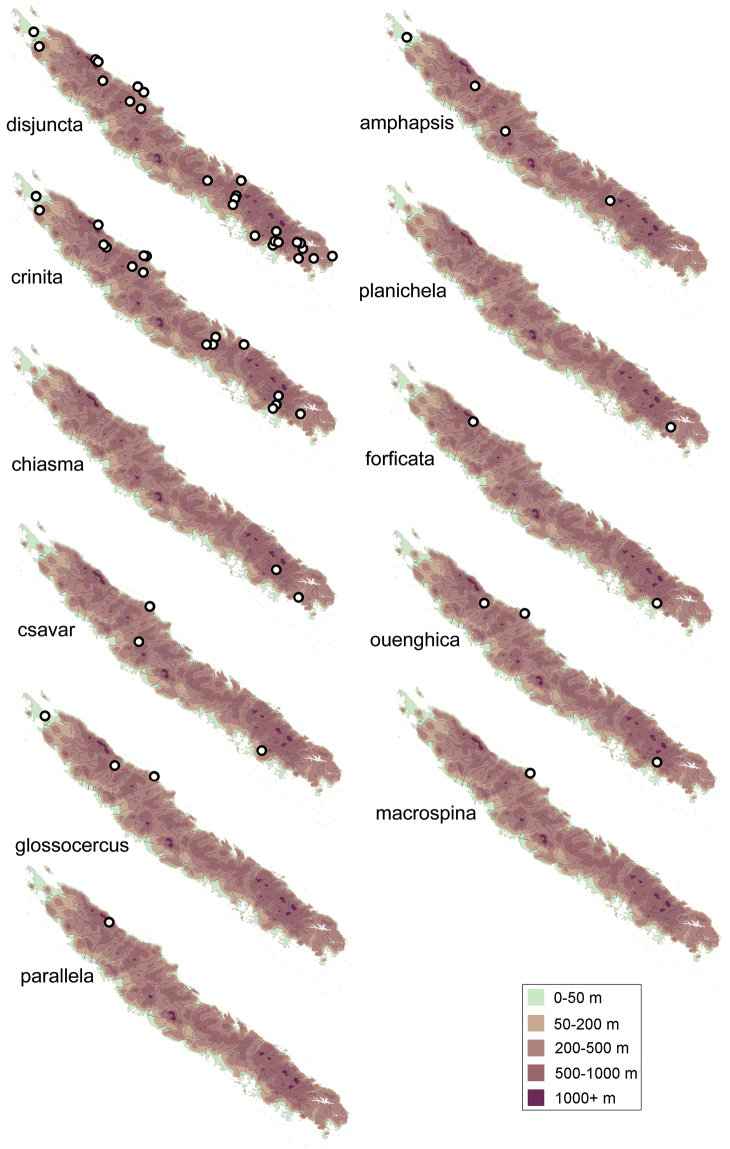
Maps of New Caledonia showing the collection sites for different species of *Acritoptila*.

### Checklist of New Caledonian *Acritoptila* species

*Acritoptila amphapsis* Kelley, 1989

*Acritoptila chiasma* Kelley, 1989

*Acritoptila crinita* Kelley, 1989

*Acritoptila karika* Oláh & Johanson, 2010a, syn. n.

*Acritoptila csavar* Oláh & Johanson, 2010a

*Acritoptila disjuncta* Kelley, 1989

*Acritoptila forficata* sp. n.

*Acritoptila glossocercus* Kelley, 1989

*Acritoptila macrospina* sp. n.

*Acritoptila ouenghica* Wells, 1995

*Acritoptila parallela* sp. n.

*Acritoptila planichela* Kelley, 1989

### Key to males of New Caledonian species of *Acritoptila* Wells

**Table:** 

1	Genitalia lacking discrete parameres; margins of abdominal segment X sclerotized (Fig. 20)	*Acritoptila ouenghica* Wells
–	Genitalia including a pair of spiny parameres; parameres simple and unbranched (Figs 3, 4) or forked (Figs 17, 18)	2
2	Gonopods fused, in form of an elongate, anteriorly directed heavily sclerotized tongue-like process (Fig. 11)	*Acritoptila glossocercus* Kelley
–	Gonopods not in form of tongue-like process, may be discrete or fused and lobe- or plate-like, membranous or sclerotized	3
3	Gonopods fully or partially sclerotized, in ventral view usually appearing as a spherical, berry-like structure (Figs 3, 5, 8) or conical lobes (Fig. 1); parameres not forked	4
–	Gonopods not sclerotized (Figs 17, 18), may bear paired small knob-like setae (Fig. 17); in ventral view parameres forked (Figs 17, 18) or forceps-like (Fig. 15) or stoutly leaf-like and asymmetrical (Fig. 23)	7
4	Parameres dilated subapically, then abruptly constricted (Figs 1, 2)	*Acritoptila disjuncta* Kelley
–	Parameres forming simple, straight to smoothly curved spines (Figs 3, 5, 8)	5
5	Gonopods fused, in ventral view in form of sclerotized spherical structure; subgenital processes broadly triangular; parameres whip-like, straight or gently bowed; setose lobes lateral to tergite X (Figs 3, 4)	*Acritoptila crinita* Kelley
–	Gonopods fused, forming rounded or ovoid fully or partially sclerotized structure; subgenital processes tongue-like, angled mesad; parameres slender and sinuous; without setose process lateral to tergite X	6
6	Apico-lateral spines on tergite X strongly curved dorsad in lateral view (Figs 9, 10)	*Acritoptila csavar* Oláh & Johanson
–	Apico-lateral spines on tergite X gently curved ventrad in lateral view (Figs 6, 7)	*Acritoptila chiasma* Kelley
7	Parameres forked	8
–	Parameres simple, not forked	9
8	Lateral branch of fork on parameres (Fig. 18) more slender than mesal branch; gonopods lacking small sclerotized knobs (rounded setae)	*Acritoptila forficata* sp. n.
–	Lateral branch of parameres (Fig. 17) more slender than mesal branch; gonopods each bearing small sclerotized knob	*Acritoptila planichela* Kelley
9	Parameres in ventral view sinuous, thread- or whip-like (Figs 14)	*Acritoptila parallela* sp. n.
–	Parameres in ventral view not thread- or whip-like, angled mesad (Figs 15, 23), down-turned in lateral view (Figs 16, 21)	10
10	Parameres asymmetrical, stout, leaf-like; gonopods triangular, each with two knob-like setae subapically (Figs 21–23)	*Acritoptila macrospina* sp. n.
–	Parameres symmetrical, in ventral view angled mesad, forceps-like; gonopods broadly rounded to subrectangular (Figs 15, 16)	*Acritoptila amphapsis* Kelley

## Supplementary Material

XML Treatment for Acritoptila

XML Treatment for Acritoptila disjuncta

XML Treatment for Acritoptila crinita

XML Treatment for Acritoptila chiasma

XML Treatment for Acritoptila csavar

XML Treatment for Acritoptila glossocercus

XML Treatment for Acritoptila parallela

XML Treatment for Acritoptila amphapsis

XML Treatment for Acritoptila planichela

XML Treatment for Acritoptila forficata

XML Treatment for Acritoptila ouenghica

XML Treatment for Acritoptila macrospina
